# HMB-45 negative angiomyolipoma of the orbit: a case report and review of the literature

**DOI:** 10.1186/s12886-016-0185-5

**Published:** 2016-01-11

**Authors:** Che-Yu Lin, Chieh-Chih Tsai, Hui-Chuan Kau, Wei-Kuang Yu, Shu-Ching Kao, Catherine Jui-Ling Liu

**Affiliations:** Department of Ophthalmology, Taipei Veterans General Hospital, No. 201, Sec.2, Shih-Pai Road, Taipei, 11217 Taiwan R.O.C.; Department of Ophthalmology, School of Medicine, National Yang-Ming University, Taipei, Taiwan; Department of Ophthalmology, Koo Foundation Sun Yat-Sen Cancer Center, Taipei, 112 Taiwan

**Keywords:** Orbit, Angiomyolipoma, Perivascular epithelioid cell tumors, HMB-45

## Abstract

**Background:**

Angiomyolipoma is a benign mesenchymal tumor composed of variable amounts of smooth muscle, adipose tissue and thick-walled blood vessels, and usually named PEComas (perivascular epithelioid cell tumors). PEComas share overlapping histopathological features with epithelioid cells along a perivascular distribution and characteristic immunohistochemistry with coexpression of myoid and melanocytic markers (HMB-45 /or Melan-A). We report the first case of primary orbital angiomyolipoma with negative melanocytic marker.

**Case presentation:**

An 80-year-old Asian woman had a 2-year history of progressive swelling in the left upper eyelid. External examination revealed 3 cm of relative proptosis of the left eye and a palpable mass in the left superonasal orbit. Computed tomographic scan demonstrated a circumscribed, heterogeneous orbital mass. Excision biopsy was done and the histological finding demonstrated the orbital mass was composed of mature adipocytes, intermingled with spindle or oval-shaped cells, and accompanied by thick-walled blood vessels. Immunohistochemically, tumor cells were positive for CD34 and HHF-35, but negative for cytokeratin, HMB-45 and Melan-A. The diagnosis of angiomyolipoma was made. No recurrence was noted at 2-year follow-up.

**Conclusion:**

In our case, the HMB-45 negativity may be explained by the rarity of the epithelioid cells, and the HMB-45 positivity is often weaker or absent in spindle cells. Angiomyolipoma, although rare, should be added to the differential diagnosis of space-occupying orbital lesion.

## Background

Angiomyolipoma, originally thought to be a hamartoma, is a benign mesenchymal tumor composed of variable amounts of smooth muscle, adipose tissue and thick-walled blood vessels, and usually named PEComas (perivascular epithelioid cell tumors). It occurs most commonly in the kidney as a sporadic case or as part of the tuberous sclerosis complex [1]. We presented the first case of primary orbital angiomyolipoma with negative melanocytic markers.

## Case presentation

**Fig. 1:**
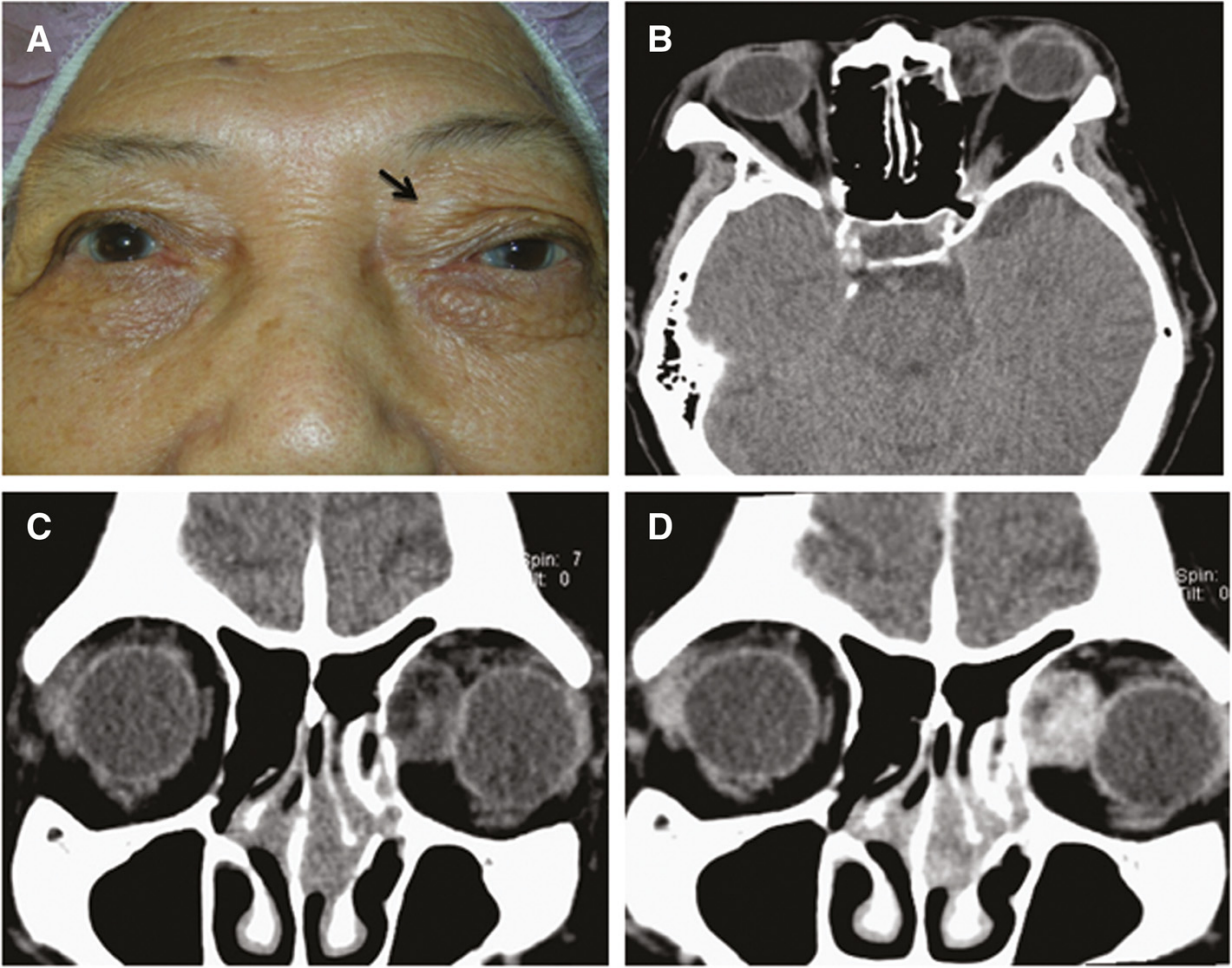
Preoperative photograph shows a palpable mass on left superonasal orbit (**a**, *arrow*). Axial (**b**) and coronal (**c**) planes of computed tomographic scan show a circumscribed soft tissue mass on the left superonasal orbit with heterogenic density containing areas of fat attenuation. The mass demonstrates heterogenous enhancement (**d**)

**Fig. 2:**
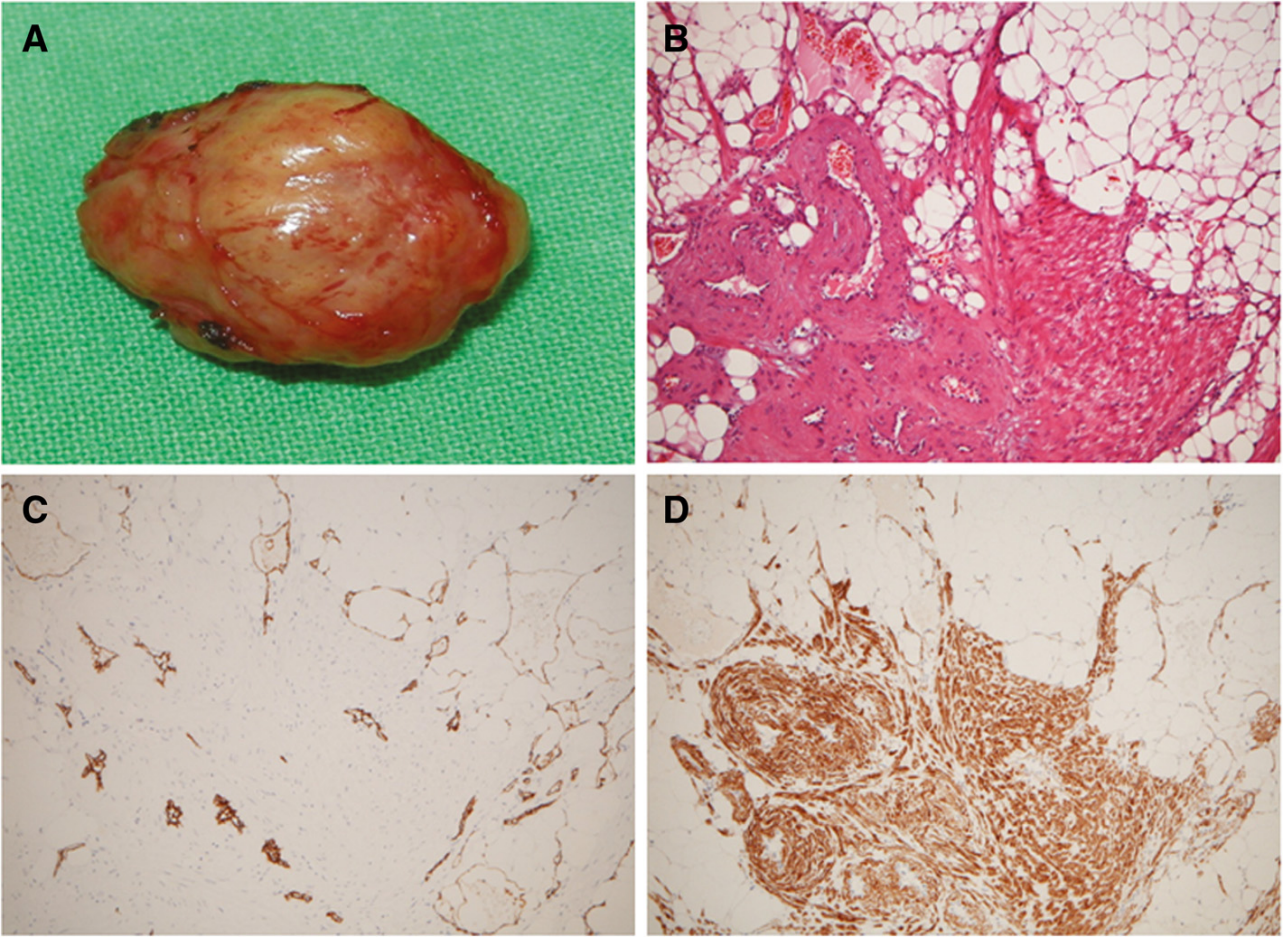
Grossly, the tumor is a 1.8 × 1.8 × 1.3 cm yellowish, encapsulated, solid mass (**a**). Microscopic features of the tumor shows it is comprised of spindle to ovoid-shaped muscle cells interspersed with irregular vascular channels and mature adipocytes (**b**, hematoxylin and eosin stain, original magnification, ×100). Immunohistochemical staining reveals that the proliferating vessels are positive for CD34 (**c**) and smooth muscle cells are positive for HHF-35 (**d**) (original magnification, ×100)

An 80-year-old woman had a 2-year history of progressive fullness in the left upper eyelid. External examination revealed 3 mm of relative proptosis of the left eye and a nontender palpable firm mass in the left superonasal orbit (Fig. 1a). The remainder of the ocular examination was within normal limit. Past medical history was otherwise unremarkable. Computed tomographic scan demonstrated a circumscribed, heterogeneous orbital mass displacing the left globe laterally (Fig. 1b, c and d). Surgical removal of the tumor was performed through anterior orbitotomy in an en bloc fashion. At the time of surgery, the 1.8 × 1.8 × 1.3 cm yellowish mass was encapsulated and solid (Fig. 2a). Histologically, the orbital mass was composed of mature adipocytes, intermingled with spindle or oval-shaped cells with eosinophilic cytoplasm, accompanied by thick-walled blood vessels (Fig. 2b). Immunohistochemically, tumor cells were positive for CD34 and HHF-35 (Fig. 2c and d), but negative for cytokeratin, HMB-45 and Melan-A. These findings confirmed the diagnosis of angiomyolipoma. Systemic check-up was unremarkable. No recurrence was noted at 2-year follow-up.

Primary orbital angiomyolipoma is a rare entity of orbital tumor. Until now, only 4 cases of ocular perivascular epithelioid cell tumor (PEComa) have been reported, and all had positive melanocytic markers [2–4]. All reported 4 cases of ocular PEComas were female and their tumor location was eyelid (2 cases), ciliary body (1 case), and orbit (1 case) respectively. PEComas often share overlapping histopathological features with epithelioid cells along a perivascular distribution and characteristic immunohistochemistry with coexpression of myoid and melanocytic markers (HMB-45 /or Melan-A) [5]. Current case is unique in that the tumor lacked reactivity for melanin-associated antigens HMB-45 and Melan-A, which is similar to some angiomyolipomas from skin, head and neck [6–9]. The HMB-45 negativity may be explained by the rarity of the epithelioid cells in these cases, and the HMB-45 positivity is often weaker or absent in spindle cells [9]. In addition, these angiomyolipomas are usually relative small, contrary to what happens to kidney and liver tumors, which are often large. However, because of the small number of reported cases, whether these HMB-negative angiomyolipoma is a new variant of PEComas require further investigation. Differential diagnosis should include giant cell angiofibroma which is a highly vascular tumor comprising a spindle-cell proliferation with numerous multinucleated giant cells and pseudovascular spaces, and immunohistochemically positive for CD34, CD99, and vimentin [10].

Approximately one third of renal angiomyolipomas occur in patients with tuberous sclerosis. However, this association has been rarely reported in extrarenal angiomyolipoma, including of ocular angiomyolipoma. Because most angiomyolipomas contain varied amounts of adipose tissue, image features of fat attenuation at unenhanced CT may help in diagnosis. Although most angiomyolipomas show a benign course, some reports have suggested that histologically atypical angiomyolipomas are potentially malignancy. Therefore, wide excision and regular follow-up are warranted.

## Conclusion

In summary, we report a case of primary orbital angiomyolipoma, which showed different immunohistochemical features from prior reported ocular PEComa. Although rare, angiomyolipoma should be added to the differential diagnosis of space-occupying orbital lesion.

### Consent

Written informed consent was obtained from the patient for publication of this case report and any accompanying images. A copy of the written consent is available for review by the editor of this journal.

## Abbreviations

PEComas: Perivascular epithelioid cell tumors
HHF35: Muscle actin antibody
HMB-45: Melanosome specific antigen
Melan-A: Melanoma antigen
CT: Computed tomography
